# Mucosal Vaccination With Recombinant *Tm-*WAP49 Protein Induces Protective Humoral and Cellular Immunity Against Experimental Trichuriasis in AKR Mice

**DOI:** 10.3389/fimmu.2022.800295

**Published:** 2022-02-07

**Authors:** Junfei Wei, Venkatesh L. Hegde, Ananta V. Yanamandra, Madison P. O’Hara, Brian Keegan, Kathryn M. Jones, Ulrich Strych, Maria Elena Bottazzi, Bin Zhan, K. Jagannadha Sastry, Peter J. Hotez

**Affiliations:** ^1^ Texas Children’s Hospital Center for Vaccine Development, National School of Tropical Medicine, Baylor College of Medicine, Houston, TX, United States; ^2^ Department of Thoracic/Head and Neck Medical Oncology, Division of Cancer Medicine, University of Texas MD Anderson Cancer Center, Houston, TX, United States; ^3^ University of Texas MD Anderson Cancer Center UTHealth Graduate School of Biomedical Sciences, Houston, TX, United States; ^4^ Department of Biology, Baylor University, Waco, TX, United States

**Keywords:** Trichuriasis, vaccine, *Tm*-WAP49, mucosal immunity, intranasal immunization, adjuvants

## Abstract

Trichuriasis is one of the most common neglected tropical diseases of the world’s poorest people. A recombinant vaccine composed of *Tm-*WAP49, an immunodominant antigen secreted by adult *Trichuris* stichocytes into the mucosa of the cecum to which the parasite attaches, is under development. The prototype is being evaluated in a mouse model of *Trichuris muris* infection, with the ultimate goal of producing a mucosal vaccine through intranasal delivery. Intranasal immunization of mice with *Tm-*WAP49 formulated with the adjuvant OCH, a truncated analog of alpha-GalCer with adjuvanticity to stimulate natural killer T cells (NKT) and mucosal immunity, induced significantly high levels of IgG and its subclasses (IgG1 and IgG2a) in immunized mice. This also resulted in a significant reduction of worm burden after challenge with *T. muris*-infective eggs. The addition of QS-21 adjuvant to this vaccine formulation further reduced worm counts. The improved protection from the dual-adjuvanted vaccine correlated with higher serum antibody responses (IgG, IgG1, IgG2a, IgA) as well as with the induction of antigen-specific IgA in the nasal mucosa. It was also associated with the robust cellular responses including functional subsets of CD4 T cells producing IL-4, and cytotoxic CD8 T cells expressing granzyme B. The worm reduction achieved by mucosal immunization was higher than that induced by subcutaneous immunization. Intranasal immunization also induced a significantly higher nasal mucosa-secreted antigen-specific IgA response, as well as higher functional cellular responses including CD4^+^IL4^+^ (Th1) and CD8^+^GnzB^+^ (Th2) T cells, and antigen-specific INFγ-producing T cells in both spleen and MLNs and antibody-producing B cells (CD19^+^B220^+^/B220^+^GL7^+^). Mucosal immunization further induced long-term T lymphocyte memory with increased central (CD62L^+^CD44^+^) and effector (CD62L^-^CD44^+^) memory subsets of both CD4 and CD8 T cells at 60 days after the last immunization. In summary, intranasal immunization with recombinant *Tm-*WAP49 protein induced strong protection versus murine trichuriasis. It represents a promising vaccination approach against intestinal nematodes.

## Introduction

Trichuriasis, caused by the infection with the gastrointestinal nematode *Trichuris trichiura* (human whipworm), is one of the most common neglected tropical diseases. More than 360 million people are chronically infected, resulting in 236,000 disability-adjusted life years (DALYs) lost based on Global Burden of Disease 2019 estimates (1). *T. trichiura* commonly infects people in rural subtropical and tropical areas where poverty is widespread and sanitation facilities are inadequate (2, 3). The infection causes malnutrition, diarrhea, anemia, and even rectal prolapse (4–6). The highest prevalence rates of infection occur in resource-poor regions of Southeast Asia, Sub-Saharan Africa, and the tropical regions of the Americas, where children carry the largest burden of *T. trichiura* infections (4, 7, 8). Most of these parasitized children are coinfected with other soil-transmitted helminths (STHs), further negatively impacting growth and cognitive development (9, 10).

Anthelmintic treatment is the only way to remove STHs, including *Trichuris* worms, from infected people, and mass drug administration with albendazole or mebendazole is now a primary approach for global health control. Since 2001, following the adoption of a World Health Assembly resolution, mass drug administration has been promoted by the World Health Organization (WHO) to treat STH infections in developing countries as an effort to eliminate neglected tropical diseases (11). However, so far this approach has not been effective for trichuriasis, and to some extent, for hookworm infection, due to disappointing drug efficacies, high rates of posttreatment reinfection, and potential drug resistance (12–15). Therefore, additional technologies are required, including combining albendazole with ivermectin or developing next-generation anthelminthic drugs (16). More recently, developing a preventive vaccine targeting children before exposure to helminths is being pursued as an alternative approach to complement anthelmintic chemotherapy as a more effective approach to control or eliminate STH infections (17).

To identify protective antigens for vaccine development against trichuriasis, we found that the excretory–secretory (ES) products from the adult whipworm of the mouse counterpart *T. muris* induced almost sterile immunity against *T. muris* infection in mice (18). *T. muris* ES products are secreted by a unique structure called the stichosome located at the anterior portion of the worm, into the colonic mucosa to facilitate parasitism in the host (19, 20). When immune sera from mice immunized with *T. muris* ES products were used to immunoscreen a *T. muris* adult cDNA library, 63 out of 102 positive clones recognized by the ES immune sera encoded the *Tm-*WAP protein, which belongs to the whey acid protein (WAP) family and contains several four-disulfide-bonded core domain repeats (18). The major 49kDa domain of *Tm-*WAP (*Tm-*WAP49) was expressed as a soluble recombinant protein in the yeast *Pichia pastoris* (r*Tm-*WAP49). Mice immunized subcutaneously with r*Tm-*WAP49 emulsified with Montanide ISA720 adjuvant produced significant worm reduction in a *T. muris*/AKR mouse model, associated with a Th2-dominated immune response (18). Since *T. muris* is an intestinal nematode that parasitizes in the cecum with its anterior end embedded in the mucosa, mucosal immunity plays a critical role in the expulsion of the worm from the intestine (21, 22). We, therefore, explored the mucosal immunity induced by *Tm-*WAP49 and determined its protective effect against *T. muris* in a mouse model. Our system utilized mice immunized intranasally with the antigen r*Tm*WAP49 combined with adjutant OCH, a truncated alpha-galactosylceramide (α-GalCer) analog that is known to induce mucosal Th2 response (23) or OCH along with QS-21 from *Quillaja saponaria* which is known to elicit both Th1 and Th2 immune response (24). Significant protection against a challenge with *T. muris*-infective eggs was observed in mice immunized intranasally with *Tm*-WAP49 using either formulation; it was associated with TH_2_-biased immune responses and secretory IgA production, indicating that mucosal immunity plays an important role in immunity against *Trichuris* infection. We also found that these studies provide a foundation for developing mucosal vaccination approaches for human helminth infections.

## Materials and Methods

### Ethics Statement

All study procedures were approved by the Institutional Animal Care and Use Committee of Baylor College of Medicine (Assurance D16-00-475) and performed in strict compliance with The Guide for the Care and Use of Laboratory Animals (8^th^ Edition) (25).

### Animal Model

The *T. muris* life cycle was maintained in susceptible STAT6K/O mice (male 6 weeks old purchased from Jackson Labs, Bar Harbor, ME, USA). *T. muris* adult worms were collected from the cecum of STAT6K/O mice 42 days postinfection, and the eggs were collected from adult female worms by culturing them in RPMI-1640 medium without serum overnight. The collected eggs were kept in water and allowed to embryonate at room temperature in the dark for 8 weeks. For the vaccine trial, 4–6-week-old male AKR mice were purchased from Jackson Labs (Bar Harbor, ME) and each animal was infected with 300 embryonated infective eggs by oral gavage in a total volume of 100 µl.

### Production of Recombinant *Tm*-WAP49 Protein

The recombinant *Tm-*WAP49 (r*Tm-*WAP49) protein was expressed in *Pichia pastoris* X-33 as described previously (18). Briefly, DNA coding for *Tm-*WAP49 was cloned into the yeast expression vector pPICZαA and then transformed into *P. pastoris* X-33. The r*Tm-*WAP49 with a His-tag at its C-terminus was expressed as soluble protein under the induction of 0.5% methanol and then purified by immobilized metal affinity chromatography. The purified protein was concentrated using Vivaspin 20 centrifugal concentrators (Sartorius, Stonehouse, UK).

### Immunization and Challenge Infection

**Figure 1:**
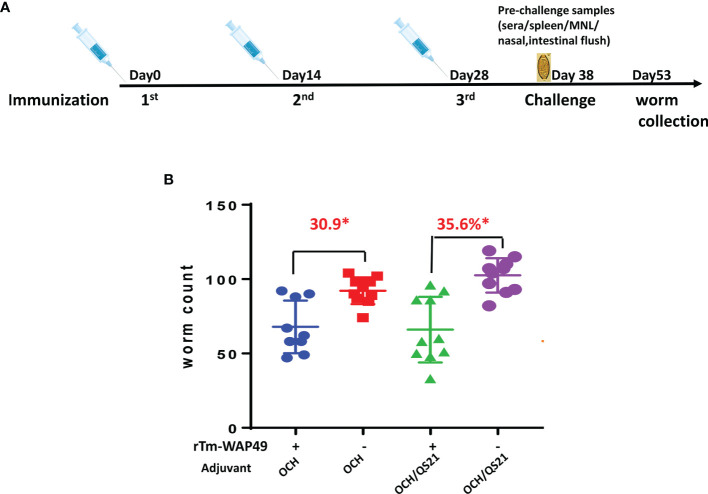
The first vaccine trial employing r*Tm-*WAP49 intranasal immunization against *T. muris* infective egg challenge. **(A)** Immunization regime. **(B)** Worm reduction in the cecum 15 days post challenge with 300 *T. muris* embryonated eggs. *p < 0.05.

Two vaccine trials with r*Tm-*WAP49 in the AKR/*T. muris* mouse model were performed. In the first trial (**Figure 1A**), 15 male 4–6-week-old AKR mice were each immunized intranasally with 100 µg r*Tm-*WAP49 formulated with 2 µg OCH (AdipoGen, Liestal, Switzerland) as an adjuvant in a total volume of 10 µl under anesthesia of isoflurane inhalation. Another group of AKR mice was intranasally immunized with the same amount of r*Tm-*WAP49 formulated with both OCH (2 µg) and 10 µg QS-21 (*Quillaja saponaria*, Creative Biolabs, Shirley, NY, USA). The other two groups of mice received the adjuvants alone as controls. All mice were boosted twice with the same dose of r*Tm-*WAP49 at 2-week intervals (on days 14 and 28). Ten days after the last immunization, tail blood was collected from each mouse and serum was separated and frozen at -20°C until used for measuring serological antibody titers. Five mice from four groups were sacrificed; the nasal lavage was collected by flushing the nasal tracts from the trachea twice with the same 500 µl PBS. To collect intestinal mucosal supernatants, a 5-cm-long intestinal section starting from the stomach was collected, opened with scissors, and soaked in 1 ml PBS for 1 h. The mucosa supernatants were collected by centrifuging at 1,000 × g, 4°C for 10 min. The nasal wash and intestinal mucosa supernatants were used to measure antigen-specific secretory IgA molecules. Spleens and mesenteric lymph nodes (MLNs) were collected for testing cellular immune responses. The remaining 10 mice from the four groups were orally challenged with 300 embryonated *T. muris* eggs in a total volume of 100 µl. Fifteen days after the challenge, all infected mice were sacrificed and their ceca were collected. Juvenile worms were collected and counted from the cecum under a microscope. The reduction in the cecum worm burden was calculated for each immunized group and compared to the worm number collected from the corresponding adjuvant control group.

**Figure 2:**
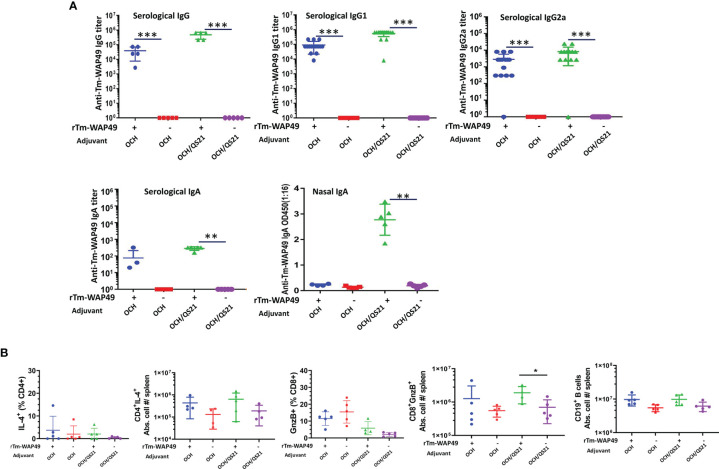
Pre-challenge antibody responses and induction of cellular immune responses by the *Tm*-WAP vaccine in mice during the first vaccine trial. **(A)** Sera and nasal lavage liquid were collected from mice intranasally immunized with r*Tm*-WAP49 formulated with OCH, or OCH/QS-21; antigen-specific IgG, IgG1, IgG2a, and IgA were measured in sera and secretory IgA measured in nasal lavage liquid by ELISA. **(B)** For cellular responses, groups of mice were euthanized pre-challenge, and spleen cells were analyzed by multiparametric flow cytometry. The total viable cells per spleen were enumerated by Trypan blue dye exclusion, and the absolute cell numbers for each lymphocyte population per mouse spleen were calculated based on the frequency of respective cell subsets and the total number of viable cells per spleen (spleen cellularity). Frequencies and absolute cell numbers of IL-4 producing CD4 T cells, granzyme B (GnzB)-expressing CD8 T cells, and absolute cell numbers of B cells in spleens of mice are shown. The data represent mean ± SD from n = 4–5 mice per group. *p < 0.05, **p < 0.01, ***p < 0.001.

To confirm the protective immunity induced by intranasal immunization and determine the duration of the mucosal immunity, a second trial was performed in AKR mice. A total of 75 4–6-week-old AKR mice were divided into 5 equal groups. Two groups of mice were each intranasally immunized with 100 µg r*Tm-*WAP49 formulated with 2 µg OCH and 10 µg QS-21 in a total volume of 12 µl under anesthesia of isoflurane inhalation. Another group of AKR mice was subcutaneously immunized with the same amount of r*Tm-*WAP49 formulated with Montanide ISA 720 (Seppic, Cologne, Germany) by emulsification at 30/70 (v/v) in a total volume of 100 µl per mouse. The other two groups of mice received QS-21/OCH adjuvants intranasally or Montanide ISA 720 emulsified with PBS subcutaneously as controls. All mice were immunized using the same regime as in the first trial, being challenged 10 days after the last immunization except for one group that was kept 60 days after the last immunization to measure the extended protective immunity. Pre-challenge samples were collected as the first trial. Fifteen days after infection, all infected mice were sacrificed and worms were collected from the cecum to determine the worm reduction in the immunized groups compared to the corresponding adjuvant control groups (**Figure 2A**).

### ELISA

Sera from all blood samples were isolated and frozen at -20°C. *Tm-*WAP49-specific IgG and the isotypes (IgG1, IgG2a) were measured in each serum sample using a modified indirect enzyme-linked immunosorbent assay (ELISA). Briefly, for measuring antibody titers in serum samples, 96-well Nunc-Immuno MaxiSorp plates (Thermo Scientific, Waltham, MA) were coated with 100 µl r*Tm-*WAP49 at a concentration of 0.75 µg/ml in coating buffer (KPL, Milford, MA) overnight at 4°C. The coated plates were then blocked overnight with 0.1% BSA in PBST (PBS + 0.05% Tween-20), then incubated with serum samples diluted in 0.1% BSA in PBST for 2 h at room temperature. The plates were washed 4 times with PBST, then incubated with diluted horseradish peroxidase (HRP)-conjugated goat anti-mouse IgG (1:6,000), IgG1 (1:4,000), or IgG2a (1:4,000) (Lifespan Biosciences, Seattle, WA) for 1 h at room temperature. The plates were washed 5 times, and subsequently, 100 µl of 4°C SureBlue TMB (KPL, Milford, MA) was added per well. The reaction was terminated by adding 100 μl 1 N HCl. The absorbance was measured at 450 nm using a spectrophotometer (BioTek, Winooski, VT).

### Cytokine Analyses

Cells were isolated from MLN by mechanical disruption by passing through a 70-micron strainer and from the spleen by mechanical disruption followed by red blood cell lysis. Cells (2 × 10^5^/well) were stimulated with 10 μg/ml r*Tm*-WAP49, 50 μg/ml *Trichuriasis* excretory-secretory extract (ES), or media in a 96-well plate by culturing for 48 h. Cell culture supernatants were analyzed by ProcartaPlex (Thermo Fisher, Waltham, MA, USA) custom Multiplex Luminex immunoassay for IL-5, IL-6, and IL-13 cytokines. The cytokine concentrations were assayed according to the manufacturer’s protocol and based on the standard curve.

### ELISpot Assay for Cytokine-Producing Cells

Cells isolated from spleen and MLN of immunized mice were subjected to ELISpot analysis for enumerating antigen-specific IFN-γ-producing cells using capture primary antibody precoated plates and reagent kit from Mabtech (Cincinnati, OH). Briefly, 200,000 cells/well were cultured in triplicates for each treatment in 96-well ELISpot plate strips for 48 h. The cells were stimulated with 2 μg/well r*Tm*-WAP49, 10 μg/well ES, or media. After another 48 h of culture, the plates were developed by sequential incubations with biotinylated secondary antibody and streptavidin-coupled alkaline phosphatase followed by chromogenic substrate BCIP/NBT-plus. The spots, representing individual IFN-γ-producing cells as spot-forming cells (SFC), on the membrane were enumerated using an automated ELISpot reader (Mabtech) and plotted as SFC/10^6^ input cells. Responses were considered positive when they were above 50 SFC/well and at least double the number obtained in cells cultured with medium alone. Antigen-specific responses were plotted after subtracting media negative control values.

### Flow Cytometry

The immune cell phenotyping and analysis of functional markers were performed by multiparametric flow cytometry analysis. Briefly, cells isolated from the spleen following RBC lysis were incubated at 37°C, 5% CO_2_ for 3–4 h with brefeldin A (GolgiPlug). Cells were first blocked using mouse Fc-block (anti-CD16/32) followed by staining for surface markers. Next, cells were washed, fixed, and permeabilized using the Fix-Perm reagent kit (BD Biosciences, Franklin Lakes, NJ, USA) followed by staining for intracellular markers. The following fluorochrome-conjugated anti-mouse antibodies were used: anti-CD11c APC, anti-CD19 BV605, anti-CD44 FITC, anti-CD62L APC-Cy7, anti-granzyme B BV421, anti-IFN-γ BV711, anti-IL4 PE, anti-B220 BUV737, anti-CD3 BV650, anti-CD4 BV786, anti-CD8 BUV396, anti-Foxp3 PE-CF594, and anti-GL7 PerCp-Cy5.5 (BD Biosciences). FACS data acquisition was done on a five-laser Fortessa X-20 flow cytometer (BD Biosciences) and analyzed using FlowJo version 10 (FlowJo LLC, Ashland, OR). Forward and side scatter parameters were used to set singlets and leukocyte gates. Fixable viability stain BV510 included in the surface antibody cocktail was used to gate out dead cells and analyze only viable cells. Overall gating strategy is shown (**Supplemental Figure S1**).

### Statistical Analysis

All data were compared by analysis of variance (ANOVA) using SPSS 13.0 software. Each vaccine and control group were compared using a two-sample t-test or Wilcoxon rank-sum test (Mann–Whitney). Data were expressed as means ± standard deviation. *p< *0.05 was considered to be statistically significant.

## Results

### Intranasal Immunization of r*Tm*WAP49 Induced Significant Protection Against *T. muris* Infection in AKR Mice

To determine the protective immunity induced by intranasal immunization with r*Tm-*WAP49, in the first trial, mice were immunized intranasally with r*Tm-*WAP49 formulated with OCH, or with OCH and QS-21. Juvenile adult worms were collected from the cecum 15 days after the challenge with 300 infective *T. muris* eggs. The results showed that mice immunized intranasally with r*Tm-*WAP49 formulated with OCH produced 30.9% worm reduction compared to control mice that had received OCH only (*p* < 0.05), while mice immunized with r*Tm-*WAP49 formulated with OCH/QS-21 produced a significantly higher worm reduction rate (35.6%) over the dual-adjuvanted control group (*p* < 0.05, **Figure 1**).

**Figure 3:**
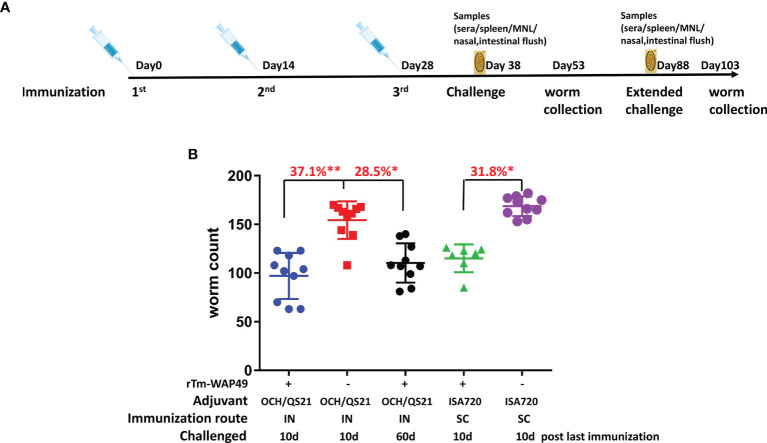
The second vaccine trial employed r*Tm-*WAP49 intranasal immunization against challenge with *T. muris* infective eggs. **(A)** Immunization regime. **(B)** Worm reduction in the cecum 15 days post challenge with 300 *T. muris* embryonated eggs. IN, intranasal immunization; SC, subcutaneous immunization. *p < 0.05, **p < 0.01.

To confirm the protective immunity induced by intranasal immunization with r*Tm-*WAP49, in the second trial, mice were intranasally immunized with the same dose of r*Tm-*WAP49 (100 µg) formulated with both adjuvants QS-21 and OCH and compared to mice subcutaneously immunized with r*Tm-*WAP49 formulated with ISA720. The results showed a similar protection rate (37.1%) as after the first trial (35.6%) in mice immunized intranasally with r*Tm-*WAP49 formulated with OCH/QS-21, representing a significant difference compared to control mice receiving adjuvants only (*p* < 0.01). This was higher than the worm reduction induced by subcutaneous immunization with the same amount of r*Tm-*WAP49 formulated with ISA720 (31.8%) (**Figure 3**). Notably, the worm reduction rate remained at 28.5% even 60 days after the final immunization (*p* < 0.05 compared to OCH/QS-21 alone), emphasizing the longevity of intranasal immunization.

### Intranasal Immunization Induced Robust Humoral Immune Responses Including Mucosal IgA as well as CD8 T Cell Response

In the first vaccine trial, intranasal immunization with r*Tm-*WAP49 formulated with OCH elicited robust serum titers of *Tm-*WAP49-specific IgG, IgG1, and IgG2a antibodies, but mice immunized with r*Tm-*WAP49 formulated with two adjuvants, OCH and QS-21, produced even stronger titers of these antibodies. Significantly, intranasal delivery of r*Tm-*WAP49 formulated with both OCH/QS-21 also induced a high level of antigen-specific secretory IgA in the nasal duct in addition to a detectable level of systemic IgA production in sera. However, intranasal immunization with r*Tm-*WAP49 formulated with OCH only induced a detectable level of antigen-specific IgA in the sera of immunized mice, but not in the nasal mucosa. In addition, no detectable antigen-specific IgA was measured in the intestinal mucosa of any group (**Figure 2A**). Additionally, the cellular immune response revealed a significant increase in absolute cell numbers of granzyme B expressing functional CD8^+^ T cells in spleens of mice treated with rTm-WAP+OCH/QS-21 over OCH/QS-21 adjuvants alone (**Figure 2B**).

**Figure 4:**
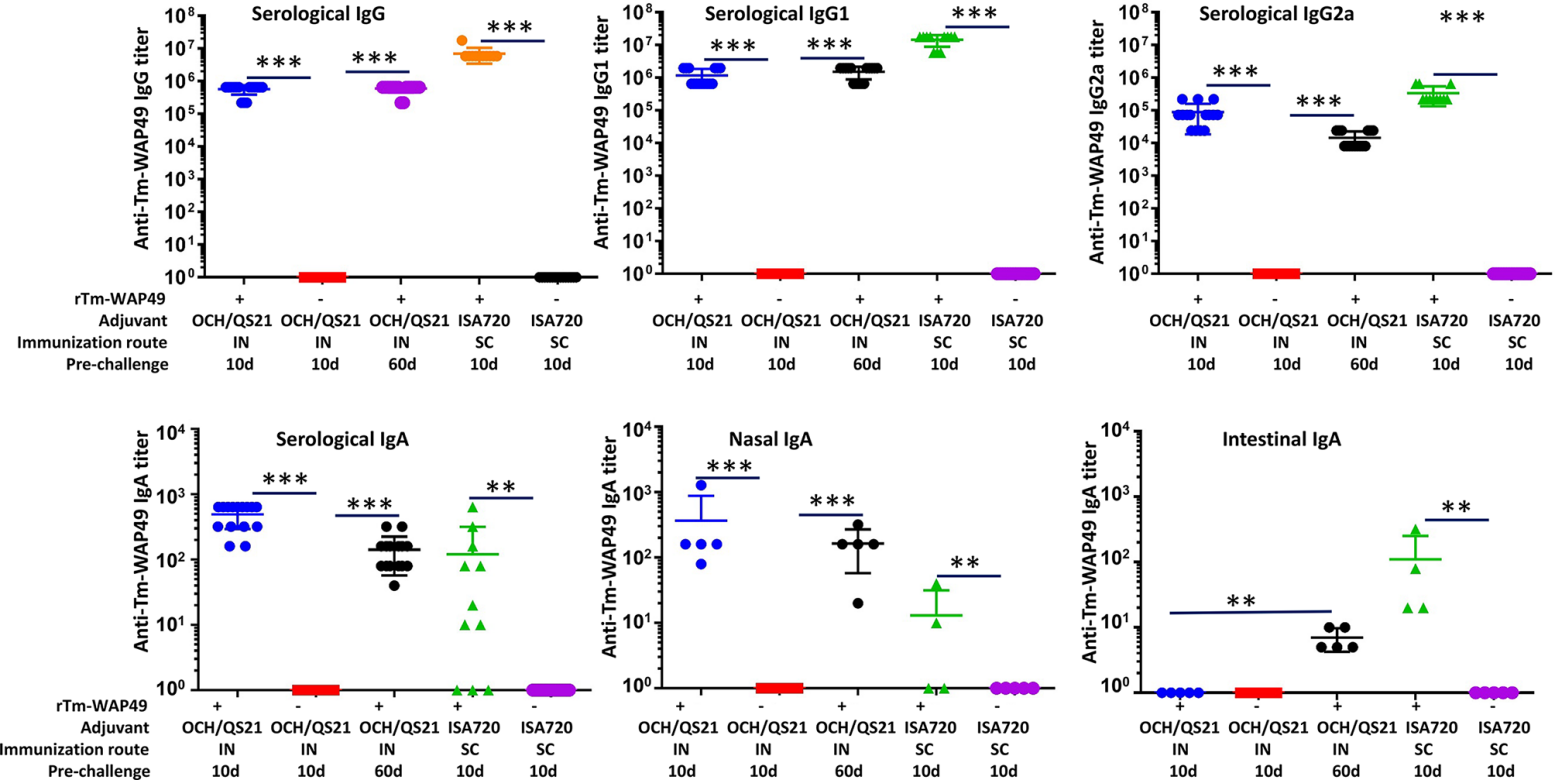
Pre-challenge humoral immune responses in mice from the second vaccine trial. Sera and nasal flush liquid were collected from mice intranasally immunized with r*Tm-*WAP49 formulated with OCH/QS-21, or subcutaneously with r*Tm-*WAP49 formulated with ISA720; antigen-specific IgG, IgG1, IgG2a, and IgA were measured in sera and secretory IgA measured in nasal flush liquid by ELISA. IN, intranasal immunization; SC, subcutaneous immunization. **p < 0.01, ***p < 0.001.

In the second vaccine trial, intranasal immunization with r*Tm-*WAP49 formulated with QS-21 and OCH elicited robust titers of *Tm-*WAP49-specific IgG (~0.5 × 10^6^), IgG1 (~1.0 × 10^6^), and IgG2a (~1 × 10^5^). The IgG and IgG1 titers remained at similar levels for up to 60 days after the last immunization; however, the anti-*Tm-*WAP49 IgG2a titer dropped about 10-fold during the same time. The *Tm-*WAP49-specific IgG, IgG1, and IgG2 titers were elevated by approximately one order of magnitude in the sera of mice immunized subcutaneously with r*Tm-*WAP49 formulated with ISA720 (**Figure 4**). Noticeably, intranasal immunization induced a significant level of antigen-specific secretory IgA in the nasal mucosa in addition to some level of antigen-specific IgA detected in sera. Interestingly, intranasal immunization with r*Tm-*WAP49 did not induce intestinal antigen-specific IgA until 60 days after the last immunization, indicating that intranasal immunization of r*Tm-*WAP49 could induce a memory response of secretory IgA in the intestine where *T. muris* worms parasitize. Subcutaneous immunization with r*Tm-*WAP49 also induced some level of intestinal IgA in addition to a low level of serological IgA. Mice receiving adjuvant only did not show any IgG and IgA responses to r*Tm-*WAP49.

### Cellular Immune Responses

**Figure 5:**
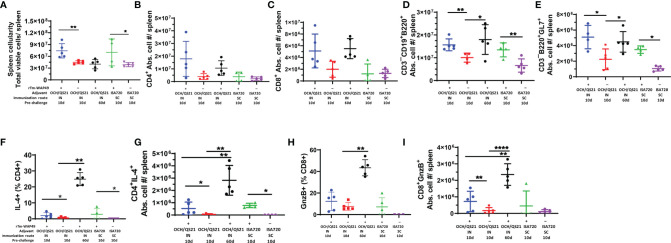
Significant induction of functional T and B cell responses by r*Tm*-WAP during the second vaccine trial. Mice were immunized with r*Tm*-WAP or adjuvant controls as described in the *Methods*. Groups of mice were euthanized pre-challenge, and spleen cells were analyzed by multiparametric flow cytometry. The total viable cells per spleen were enumerated by Trypan blue dye exclusion, and the absolute cell numbers for each lymphocyte population per mouse spleen shown were calculated based on the frequency of respective cell subsets and the total number of viable cells per spleen (spleen cellularity). **(A)** The total numbers of CD4 T cells **(B)**, CD8 T cells **(C)**, and B cells **(D)** along with the functional subsets in terms of IL-4-producing CD4 T cells **(F, G)**, granzyme B (GnzB) expressing CD8 T cells **(H, I)**, and germinal center B cell expressing GL-7 **(E)**. The data represent mean ± SD from n = 4–5 mice per group. IN, intranasal immunization; SC, subcutaneous immunization. *p < 0.05, **p < 0.01, ****p < 0.0001.

In the first vaccine trial, along with humoral immune responses, the induction of functional T cell responses was analyzed in mice immunized by the intranasal route with *Tm-*WAP49 formulated with OCH or OCH/QS-21 adjuvants (**Figure 3B**). The data indicated that CD4 T cells producing IL-4 and total B cell populations were relatively higher in *Tm-*WAP49 formulated with OCH or OCH/QS-21 compared to control groups of mice immunized with adjuvants only. Moreover, we observed that cytotoxic CD8 T cells (CTLs) expressing granzyme B were significantly increased in *Tm-*WAP49 formulated with OCH/QS-21 compared to adjuvants only controls. A more extensive analysis of cellular immune responses employing multiple phenotypic and functional markers was performed in the second vaccine trial. Significant increases in total spleen cellularity were observed in the vaccine-treated groups as opposed to their respective adjuvant controls at day 10 (**Figure 5A**). Multiparametric flow cytometry analyses showed that there were no significant differences in the absolute cell numbers for total CD4 (**Figure 5B**) and CD8 T cells (**Figure 5C**) induced by vaccination as compared to adjuvant controls. However, absolute numbers of CD3^–^CD19^+^B220^+^ total B cells (**Figure 5D**) and germinal center B cell subsets expressing specific marker GL-7 on the surface (**Figure 5E**) were significantly increased in the vaccine groups at day 10 compared to adjuvant controls. Moreover, frequencies, as well as absolute cell numbers of functional subsets of CD4 T cells producing IL-4 (**Figures 5F, G**), were significantly upregulated in vaccine-treated groups. The frequencies of cytotoxic CD8 T cells expressing granzyme B were slightly higher with WAP49 + OCH/QS-21 intranasal vaccination compared to adjuvant control (**Figure 5H**). When the frequencies were converted to absolute cell numbers, there was a significant increase in these functional subsets with WAP49 + OCH/QS-21 intranasal vaccination compared to adjuvant control vaccination at day 10 (**Figure 5I**). Interestingly, these increases in functional CD4 and CD8 T cell responses were not only sustained but further enhanced by day 60 in mice vaccinated with r*Tm-*WAP49 + OCH/QS-21 (**Figures 5D–I**). Unlike the r*Tm-*WAP49 + OCH/QS-21 intranasal vaccine, the r*Tm-*WAP49 + ISA720 SC vaccine significantly induced functional CD4 response (CD4^+^IL-4^+^), but not functional CD8 response (CD8^+^GnzB^+^) relative to ISA720 adjuvant control (**Figures 5H, I**).

**Figure 6:**
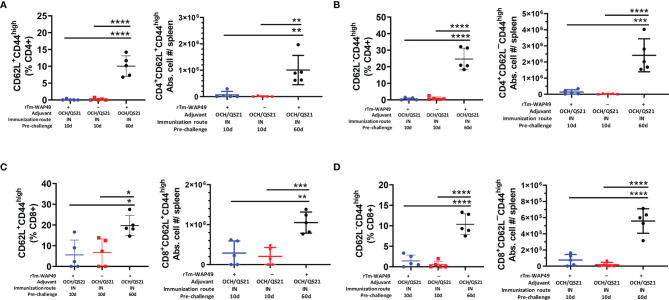
Mucosal immunization with the Tm-WAP vaccine induces long-term T lymphocyte memory response. Pre-challenge spleen samples were analyzed by flow cytometry for T cell memory based on CD62-L and CD44 expression. The CD62L+CD44+(high) central memory (T_CM_) CD4 **(A)** and CD8 **(B)**, as well as the CD62-L-CD44+(high) effector memory (T_EM_) CD4 **(C)** and CD8 **(D)** T lymphocyte frequencies and absolute cell numbers in three treatment groups as indicated, are shown. The data represent mean ± SD from 5 (n) mice per group. IN, intranasal immunization. ANOVA with Tukey *post-hoc* test, *p < 0.032 **p < 0.0021, ***p < 0.0002, ****p < 0.0001.

Importantly, in agreement with the observed humoral immune responses (**Figures 2A**, **4**), the total frequencies of B cells (CD19^+^B220^+^) as well as the functional germinal center B cell subsets (B220^+^GL7^+^), important for antibody production, were significantly higher in mice receiving the intranasal vaccine formulated with OCH/QS-21 or after the systemic subcutaneous immunogen formulated with ISA720 (**Figures 5D, E**). The enhanced total B cell and functional germinal center B cell responses were also found to be sustained at day 60 in mice receiving intranasal r*Tm*-WAP49 + OCH/QS-21 vaccine formulation. We also observed robust induction of central memory (CD62L^+^C44^High^) CD4 (**Figure 6A**) and CD8 (**Figure 6C**) T cells as well as effector memory (CD62L¯CD44^High^) subsets of CD4 (**Figure 6B**) and CD8 (**Figure 6D**) T cells 60 days after intranasal vaccination with r*Tm*-WAP49 + OCH/QS-21.

**Figure 7:**
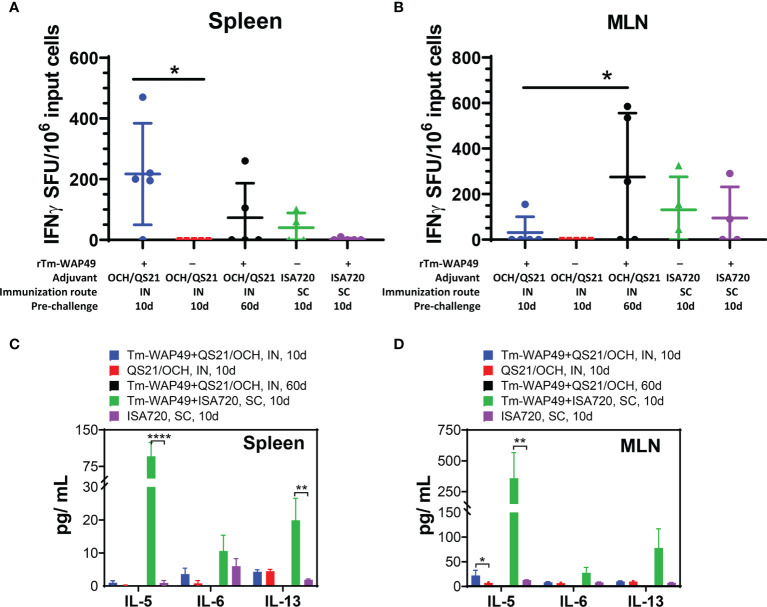
Mucosal immunization with the Tm-WAP vaccine induces antigen-specific T cells and cytokine secretion. Pre-challenge spleen and mesenteric lymph node (MLN) cells were stimulated with TmWAP49 antigen or media in triplicate wells for 48 h in ELISPOT plates, developed, and analyzed for IFN-γ+ spots. The antigen-specific IFN-γ spot-forming units (SFU) after subtracting spots from media control wells for each sample are shown for spleen **(A)** and MLN **(B)**. The data represent mean ± SD from n = 4–5 mice per group. Aliquots of 48-h culture supernatants were analyzed for various cytokines by multiplex Luminex assay. The antigen-specific secretion of cytokines is shown for spleen **(C)** and MLN **(D)** after subtracting the values for media controls for each sample. The data represent mean ± SD from n = 4–5 mice per group. IN, intranasal immunization; SC, subcutaneous immunization. *p < 0.05, **p < 0.0021, ****p < 0.0001.

Analysis of cytokine-producing cells by the ELISpot assay interestingly revealed significant populations of cells producing IFN-γ in the spleen (**Figure 7A**) and MLN (**Figure 7B**) of mice vaccinated intranasally with r*Tm*-WAP49 + OCH/QS-21, but not in mice vaccinated with ISA720 formulation *via* the subcutaneous route, suggesting a stronger Th1 response with the intranasal vaccine formulated with OCH/QS-21. This along with a significant increase in IL-5 detected in the Luminex multiplex cytokine assay in the culture supernatants of MLN (**Figure 7D**) suggests a likely more balanced Th1/Th2 response with r*Tm*-WAP49 + OCH/QS-21 vaccine, but significantly higher levels of IL-5 from spleen (**Figure 7C**) and MLN cells (**Figure 7D**), and IL-13 in spleen from mice that received vaccine formulated with ISA720 and stimulated *ex vivo* with r*Tm-*WAP49 (**Figure 7D**), but the absence of IFN-γ-producing cells in the ELISpot assay indicates a predominantly Th2 response with the subcutaneous formulation.

## Discussion

*Tm-*WAP49 is an immunodominant antigen secreted by adult *T. muris* stichocytes into the mucosa of the cecum to which the *Trichuris* parasite attaches (18). Secretion of *Tm-*WAP49 into the intestinal epithelium plays an essential role in the parasitism of the nematode possibly through inducing pore formation in lipid bilayers of the mucosa to facilitate the invasion of the worm’s anterior section into the cecal mucosa (26). Alternatively, this antigen may function as a natural immunomodulator to attenuate host immune responses (18, 27). These essential roles in whipworm parasitism suggest a potential of *Tm-*WAP49 as a trichuriasis vaccine candidate. Indeed, subcutaneous immunization with *Tm-*WAP49 induced significant protection against *T. muris* infection in mice which was associated with Th2-predominant immune responses (18). *Tm-*WAP49 is the first *Trichuris*-secreted individual protein identified as a vaccine candidate that provides protective immunity against *Trichuris* infection.

Host mucosal immune responses are essential for protective immunity against STHs in general and especially *Trichuris* sp (28, 29). These findings suggest the potential of mucosal delivery for *Trichuris* vaccines, which would represent a distinct advantage for immunizing populations in resource-poor settings. To improve the protective immunity of *Tm-*WAP49 against *Trichuris* infection, we immunized mice with *Tm-*WAP49 *via* the intranasal route combined with the adjuvant OCH, a sphingosine-truncated analog of alpha-GalCer with adjuvanticity to stimulate natural killer T cells (NKT) and mucosal immunity in general (23, 30). NKT cells play an important role in regulating the differentiation of Th1 or Th2 cells by secreting different cytokines such as IFN-γ, TNF-α, and IL-4 (31). OCH activates NKT cells in the local nasal mucosa to mainly secrete IL-4 cytokines that induce antibody responses (32). Our results demonstrate that repeated nasal mucosal immunization with r*Tm-*WAP49 formulated with OCH induced significantly high levels of IgG and its subclasses (IgG1 and IgG2a) in sera and secreted IgA in the nasal mucosa of immunized mice.

Even though the Th2 immune response is crucial in protective immunity against helminth infection, more evidence showed that Th1/Th2 mixed immune responses produced better protection against helminth infections (33, 34). QS-21 is a natural saponin fraction derived from *Quillaja saponaria* Molina, an evergreen tree native to South America. QS-21 has been a highly promising adjuvant candidate in humans with extensive testing in more than 100 clinical trials due to its ease of purification, safety profile, and ability to promote adequate immune responses (35, 36). QS-21 is a component of advanced multi-adjuvant systems (AS) in recently FDA-approved prophylactic vaccines against infectious disease, namely, Herpes Zoster (Shingrix™) (37) and malaria (Mosquirix™) (38). Mice immunized with r*Tm-*WAP49 formulated with both OCH and QS-21 adjuvants induced a significantly higher serological IgG subclass response over OCH only. Moreover, the r*Tm-*WAP49 intranasal vaccine containing QS-21/OCH showed significantly higher functional CD8 CTL responses compared to QS-21/OCH only, which was not observed with vaccine formulated with OCH alone. Interestingly, intranasal immunization with r*Tm-*WAP49 formulated with OCH only induced systemic IgA, but no secreted specific IgA was observed in the nasal mucosa. However, mice immunized with r*Tm-*WAP49 formulated with both OCH and QS-21 secreted significant levels of antigen-specific IgA in the nasal mucosa, suggesting that QS-21 contributes to the induction of a robust mucosal antigen-specific humoral response. This is in agreement with the literature where QS-21 has been shown to promote both humoral and cellular immune responses (35, 36) and a balanced Th1/Th2 response (39) and OCH is known to primarily induce NKT responses (30). The challenge study after three intranasal immunizations showed that mice immunized with r*Tm-*WAP49 formulated with OCH showed 30.9% worm reduction against challenge with *T. muris* infective eggs compared to control mice that had received OCH only (*p* < 0.05). Mice intranasally immunized with r*Tm-*WAP49 formulated with OCH/QS-21 produced the highest worm reduction (35.6%). The better protection in these mice is correlated with higher serological antibody responses (IgG, IgG1, IgG2a, IgA) and cellular production, especially with induction of antigen-specific IgA in the nasal mucosa.

The significant protection against *T. muris* infection by intranasal immunization was reproduced in the second vaccine trial. Mice intranasally immunized with r*Tm-*WAP49 formulated with OCH/QS-21 showed not only significantly high titers of IgG, IgG1, IgG2a, and IgA in sera but also robust cellular responses including functional subsets of CD4 T cells producing IL-4 and cytotoxic CD8 T cells expressing granzyme B. The robust humoral responses are consistent with the finding of increased numbers of mature B cells (CD19+B220+) and germinal center B cell subsets (B220+GL7+) in spleens. The germinal center, a special microenvironment in secondary lymphoid organs, is observed mainly in response to T-cell-dependent antigen immunization. Mature B cells are known to edit their immunoglobulin genes through somatic hypermutation and class-switching recombination after entering the germinal center and then differentiate into memory and plasma cells (40, 41). We identified activated germinal center B cells based on high GL7 expression, which is a well-established marker of germinal centers in the immunized spleen or lymph nodes (42–44).

After being challenged with *T. muris*-infective eggs, mice intranasally immunized with r*Tm-*WAP49 formulated with OCH/QS-21 produced a comparable worm reduction (37.1%) to the first trial (35.6%). Worm reduction induced by mucosal immunization was slightly (+ ~5%) higher than what was achieved after subcutaneous immunization with r*Tm-*WAP49 formulated with Montanide ISA-720 (30.9%) although the latter induced even higher IgG, IgG1, and IgG2a antibody responses.

We further observed some interesting differences in humoral and cellular immune responses. The intranasal vaccine (r*Tm-*WAP49 + OCH/QS-21) induced significantly higher nasal mucosa-secreted antigen-specific IgA levels, as well as higher cellular responses including functional Th2 (CD4+IL4+) and Th1 (CD8+GnzB+) T cells and antibody-producing B cells (CD19+B220+/B220+GL7+) than the subcutaneous vaccine (r*Tm-*WAP49 + ISA-720). Mucosal immunization with the r*Tm-*WAP49 + OCH/QS-21 vaccine also induced higher antigen-specific IFN-γ-producing T cells in both spleen and MLN. Together, the results suggest that while the subcutaneous vaccine containing ISA-720 produces a predominantly Th2 response, the intranasal vaccine formulated with OCH/QS-21 elicits a more diverse immunity involving robust antigen-specific humoral as well as more balanced Th1/Th2 cellular responses.

The mucosal immune system contains mucosa-associated lymphoid tissues (MALT) in the mucosal *lamina propria* (45). Secretory IgA is the predominant antibody isotype with a polymeric structure in the mucosal immunity which is different from serological IgA in the monomeric form (46). Except for the structural difference, the serological IgA and mucosal sIgA function differently in defense immunity (47). Secreted on the surface of the mucosa, sIgA plays a crucial role in defending foreign pathogens (48) including intestinal helminths. The mechanisms underlying protective immunity in this study requires further elucidation. Parasite-specific sIgA was associated with reduced worm growth and fecundity (49) as well as worm expulsion (50). We demonstrated that intranasal mucosal immunization with vaccine candidate *Tm-*WAP49 induced strong nasal mucosal sIgA response correlated with higher worm reduction against *T. muris* infection in mice, further suggesting that *Tm-*WAP49-specific sIgA plays an important role in the protective immunity against *T. muris* infection. Even though intranasal immunization induces mainly sIgA in the nasal mucosa, the antigen-specific IgA was also observed at a lower level in intestinal mucosa in this study. This result is consistent with the observation that the intranasal mucosa acts synergistically with the intestinal or urogenital mucosa despite being anatomically separate (51). It has also been identified that sIgA can be elicited by mucosal vaccines against influenza virus (52), making mucosal vaccines more attractive for respiratory or intestinal infectious diseases (53).

In this study, we further found that intranasal immunization with *Tm-*WAP49 induced strong responses of central (CD62L^+^CD44^+^) and effector (CD62L^-^CD44^+^) memory subsets of both CD4 and CD8 at 60 days after last immunization, but not found at 10 days post immunization, indicating that memory immune response has been developed 2 months post immunization. This finding is correlated with the strong humoral (antibodies) and cellular responses in mice 2 months after last intranasal immunization with *Tm-*WAP49, especially with even stronger Th1 (CD8^+^GnzB+), Th2 (CD4^+^IL-4^+^) and maternal B cell (CD19^+^B220+ and G17^+^B220^+^) responses in mice after 2 months (**Figure 6**). The generation of immune memory is a key issue for the success of a vaccine to provide extended, adequate, and rapid protection against helminths and other pathogens (54, 55). Interestingly, intestinal mucosal sIgA was not observed until 60 days post intranasal immunization with *Tm-*WAP49, indicating that memory response is necessary for the mucosal IgA response transferred from respiratory tract mucosa to intestinal mucosa. The significant stimulation of the memory immune response after intranasal immunization rewarded the mice with prolonged protection against *T. muris* challenge.

In summary, intranasal immunization with recombinant *Tm-*WAP49 protein formulated with OCH and QS-21 adjuvants induced strong Th1 and Th2 mixed immune responses with a Th2 bias, inducing a robust mucosal secretory IgA response, which confers immunized mice with significant protection against challenge with *T. muris*-infective eggs. Mucosal immunization with *Tm-*WAP49 also induced memory immunity in immunized mice to extend the protection against *T. muris* infection up to 60 days post the last immunization. The results obtained in this study suggest that mucosal immunity through intranasal immunization is an effective vaccination approach to induce protective immunity against intestinal nematodes like *Trichuris*.

## Data Availability Statement

The original contributions presented in the study are included in the article/**Supplementary Material**. Further inquiries can be directed to the corresponding authors.

## Ethics Statement

The animal study was reviewed and approved by the Institutional Animal Care and Use Committee of Baylor College of Medicine.

## Author Contributions

PH and KS conceived and designed the study. JW, BZ, VH, BK, AY, and MO’H performed the experiments. BZ and KS wrote the manuscript. PH, US, KS, KJ, MB, and VH analyzed the data and revised the manuscript. All authors contributed to the article and approved the submitted version.

## Funding

The research was supported by NIH R21 grant: 1R21AI144555-01.

## Conflict of Interest

The authors declare that the research was conducted in the absence of any commercial or financial relationships that could be construed as a potential conflict of interest.

## Publisher’s Note

All claims expressed in this article are solely those of the authors and do not necessarily represent those of their affiliated organizations, or those of the publisher, the editors and the reviewers. Any product that may be evaluated in this article, or claim that may be made by its manufacturer, is not guaranteed or endorsed by the publisher.
