# Supplementary figures and images for: Mucosal Vaccination With Recombinant Tm-WAP49 Protein Induces Protective Humoral and Cellular Immunity Against Experimental Trichuriasis in AKR Mice
Source: Front Immunol. 2022 Feb 7;13:800295. doi: 10.3389/fimmu.2022.800295 (PMC8859434; doi:10.3389/fimmu.2022.800295)

Supplemental Figure S1

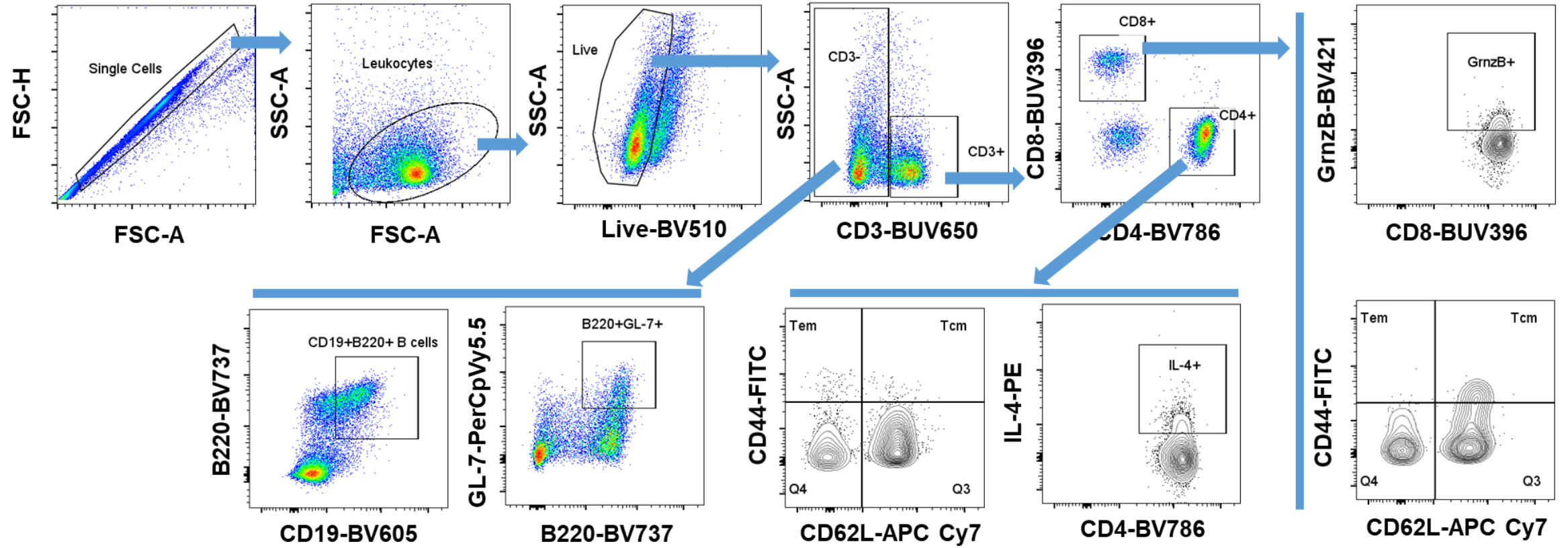

Supplement: Supplementary file 1 [file Image_1.pdf]
